# Mitral valve annular velocity measurements derived from cine MRI: validation against Doppler echocardiography

**DOI:** 10.1186/1532-429X-14-S1-W19

**Published:** 2012-02-01

**Authors:** Christoph Guetter, Paaladinesh Thavendiranathan, Marie-Pierre Jolly, Xiaoguang Lu, Hui Xue, Orlando P Simonetti

**Affiliations:** 1 Siemens Corporation, Corporate Research, Princeton, NJ, USA; 2 Dorothy M. Davis Heart and Lung Research Institute, The Ohio State University, Columbus, OH, USA; 3 Cleveland Clinic Foundation, Cleveland, OH, USA

## Summary

Assessment of mitral annular tissue velocity plays an essential role in the evaluation of diastolic dysfunction. We have previously shown that mitral annular velocities can be derived from standard four-chamber cine SSFP images by automatically detecting and tracking the mitral valve insertion points [1]. However, this method has not been validated against tissue Doppler echocardiography, the standard clinical method for evaluating diastolic function.

The objective of this study was to assess the accuracy of early and late diastolic (e’ and a’) mitral annular velocities derived from high temporal resolution SSFP cine by correlating with tissue Doppler echocardiography.

## Background

Assessment of mitral annular tissue velocity plays an essential role in the evaluation of diastolic dysfunction. We have previously shown that mitral annular velocities can be derived from standard four-chamber cine SSFP images by automatically detecting and tracking the mitral valve insertion points[1]. However, this method has not been validated against tissue Doppler echocardiography, the standard clinical method for evaluating diastolic function. The objective of this study was to assess the accuracy of early and late diastolic (e’ and a’) mitral annular velocities derived from high temporal resolution SSFP cine by correlating with tissue Doppler echocardiography.

## Methods

Nine healthy volunteers (5 males, mean age 26.7 yrs) gave informed consent to undergo CMR and echocardiography exams on the same day. Retrospectively and prospectively gated cine SSFP images in the four-chamber view were acquired during breath-hold on a 3T system (Siemens, Tim Trio). Rate 3 acceleration was used to achieve the following parameters: 17 ms true temporal resolution, 2.0x2.6mmx8mm voxel size, 12-heartbeat duration. Mitral inflow peak velocity (E) was measured using retro-gated segmented PC: TR/TE = 4.5/1.9ms, 10mm slice, 100 x 192 matrix, TSENSE rate=3, VENC=150cm/s, true temporal resolution 36ms. Trans-thoracic tissue Doppler echocardiography was used to measure mitral annular tissue velocities and inflow velocity for 3 heart beats and averaged.

**Figure 1:**
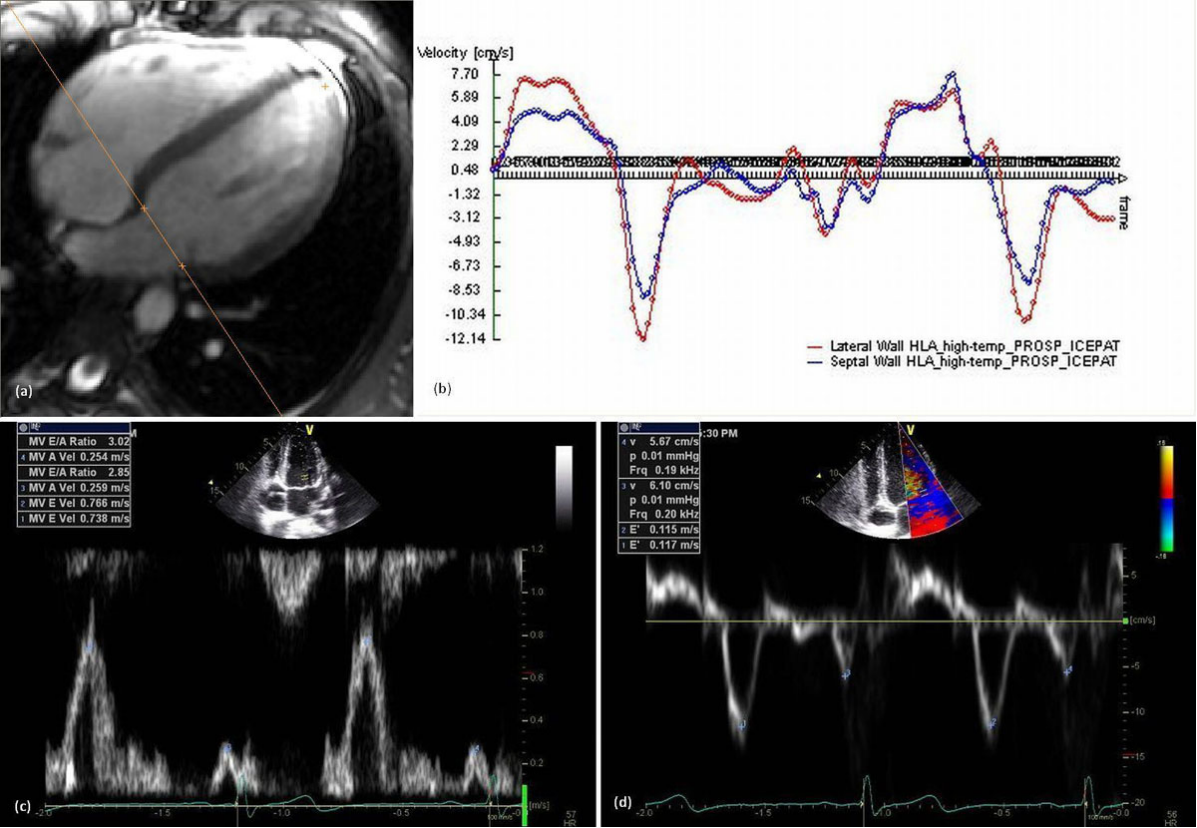
Mitral valve annulus velocity tracking in prospectively triggered MR (a), (b), mitral inflow velocity (c), and tissue velocities (d) from Doppler echocardiography. Mean lateral e’ velocities are 11.6 cm/s (echo) and 11.9 cm/s (CMR), and mean lateral a’ velocities are 5.8 cm/s (echo) and 4.9 cm/s (CMR).

Apex-to-base velocities of the medial and lateral MV insertion points were estimated from cine SSFP images by automatic detection[2] and tracking of position over the entire cardiac cycle using deformable registration[3] as shown in Figure 1. Manual corrections were applied in half of the cases where automated tracking was sub-optimal.

## Results

There was good agreement between echo and CMR mean e’ and a’ velocities and E/e’ ratios (Table 1). Concordance correlation analysis of combined lateral and medial wall measurements revealed substantial concordance between echo and prospectively triggered (0.64) and retro-gated (0.61) e’ measurements as well as between echo and prospectively triggered (0.65) E/e’ measurements. As expected, retrospectively triggered and retro-gated measurements show strong concordance ranging from 0.81 to 0.91. Although concordance in a’ velocities was poor, the utility of this parameter in clinical practice is not clear.

**Table 1:** 

	Medial		Lateral			Echocardiography		
	
Acquisition Technique	e'	a'	e'	a'	E/e'	e'	a'	E/e'
	
	mean ± std (cm/s)	mean ± std (cm/s)	mean ± std (cm/s)	mean ± std (cm/s)	mean ± std (cm/s)	Concordance Corr.	Concordance Corr.	Concordance Corr.
Retrospective CMR	11.29 ± 3.93	3.30 ± 1.34	15.98 ± 4.76	4.51 ± 1.82	5.0 ± 1.3	0.61	-0.09	0.58

Prospective CMR	11.04 ± 2.79	3.95 ± 1.60	15.31 ± 3.92	4.82 ± 1.75	5.1 ± 1.0	0.64	0.03	0.65

Echocardiography	11.62 ± 3.09	6.17 ± 0.86	14.80 ± 3.16	6.24 ± 1.59	5.0 ± 1.3	n/a	n/a	n/a

Mean e’ and a’ velocities measured at medial and lateral myocardium wall as well as combined lateral and medial E/e’ ratios and concordance correlation measured from 9 healthy volunteers.

## Conclusions

Despite the small sample size there was substantial concordance between CMR and echo measurement of e’. This suggests that mitral annular velocity can be measured accurately and extracted in an automated fashion from high temporal resolution cine MR acquired in a reasonable breath-hold time. This method combined with mitral inflow velocities offers the potential for CMR to provide important information regarding diastolic function and filling pressures.

## Funding

NIH grant RO1 HL 102450;
